# Supplementary material for: COZOID: contact zone identifier for visual analysis of protein-protein interactions
Source: BMC Bioinformatics. 2018 Apr 6;19:125. doi: 10.1186/s12859-018-2113-6 (PMC5889581; doi:10.1186/s12859-018-2113-6)
Supplement: Supplementary file 2 — Software build. Executable binary file of the software tool. (ZIP 118,784 kb) [file 12859_2018_2113_MOESM2_ESM.zip › cozoid/platform/docs/testng-6.5.1-javadoc.zip!/org/testng/ITestClass.html]

ITestClass (TestNG)


---


|  |  |  |
| --- | --- | --- |
| **Overview** \| **Package** \| **Class** \| **Tree** \| **Deprecated** \| **Index** \| **Help** | |  |
| **PREV CLASS**   **NEXT CLASS** | **FRAMES**    **NO FRAMES**     **All Classes** |  |
| SUMMARY: NESTED \| FIELD \| CONSTR \| METHOD | DETAIL: FIELD \| CONSTR \| METHOD |  |


---


## org.testng Interface ITestClass

**All Superinterfaces:**: IClass, java.io.Serializable

---

``` public interface ITestClass extends IClass, java.io.Serializable ```

This class represents a test class:

- The test methods- The configuration methods (test and method)- The class file

Note that the methods returned by instances of this class
are expected to be correct at runtime. In other words,
they might differ from what the ITestMethodFinder returned
since ITestClass will take into account the groups being
included and excluded.

**Author:**
:   Cedric Beust

---

| **Method Summary** | |
| --- | --- |
| `ITestNGMethod[]` | `getAfterClassMethods()`             Returns all the methods that should be invoked after all the tests have been run on this class. |
| `ITestNGMethod[]` | `getAfterGroupsMethods()`             Returns all @Configuration methods that should be invoked after certain groups. |
| `ITestNGMethod[]` | `getAfterSuiteMethods()`             Returns all the methods that should be invoked after the suite has run. |
| `ITestNGMethod[]` | `getAfterTestConfigurationMethods()`             Returns all @Configuration methods that should be invoked last before any others in the current test. |
| `ITestNGMethod[]` | `getAfterTestMethods()`             Returns all the methods that should be invoked after a test method completes. |
| `ITestNGMethod[]` | `getBeforeClassMethods()`             Return all the methods that should be invoked after the test class has been created and before any of its test methods is invoked. |
| `ITestNGMethod[]` | `getBeforeGroupsMethods()`             Returns all @Configuration methods that should be invoked before certain groups. |
| `ITestNGMethod[]` | `getBeforeSuiteMethods()`             Returns All the methods that should be invoked before the suite is run. |
| `ITestNGMethod[]` | `getBeforeTestConfigurationMethods()`             Returns all @Configuration methods that should be invoked before any others in the current test. |
| `ITestNGMethod[]` | `getBeforeTestMethods()`             Returns all the methods that should be invoked before a test method is invoked. |
| `int` | `getInstanceCount()` |
| `long[]` | `getInstanceHashCodes()`             TODO cquezel JavaDoc. |
| `java.lang.Object[]` | `getInstances(boolean reuse)`             Returns all the instances the methods will be invoked upon. |
| `ITestNGMethod[]` | `getTestMethods()`             Returns all the applicable test methods. |

**Methods inherited from interface org.testng.IClass**

`addInstance, getName, getRealClass, getTestName, getXmlClass, getXmlTest`

**Method Detail**

### getInstances

```
java.lang.Object[] getInstances(boolean reuse)
```

:   Returns all the instances the methods will be invoked upon.
    This will typically be an array of one object in the absence
    of a @Factory annotation.

    :   **Specified by:**: `getInstances` in interface `IClass`
    :   **Parameters:**: `reuse` - flag if a new set of instances must be returned (if set to false) **Returns:**: All the instances the methods will be invoked upon.

---


### getInstanceHashCodes

```
long[] getInstanceHashCodes()
```

:   TODO cquezel JavaDoc.

    :   **Specified by:**: `getInstanceHashCodes` in interface `IClass`

---


### getInstanceCount

```
int getInstanceCount()
```

:   **Specified by:**: `getInstanceCount` in interface `IClass`
:   **Returns:**: The number of instances used in this class. This method is needed for serialization since we don't know ahead of time if the instances of the test classes will be serializable.

---


### getTestMethods

```
ITestNGMethod[] getTestMethods()
```

:   Returns all the applicable test methods.

    :   **Returns:**: All the applicable test methods.

---


### getBeforeTestMethods

```
ITestNGMethod[] getBeforeTestMethods()
```

:   Returns all the methods that should be invoked
    before a test method is invoked.

    :   **Returns:**: All the methods that should be invoked before a test method is invoked.

---


### getAfterTestMethods

```
ITestNGMethod[] getAfterTestMethods()
```

:   Returns all the methods that should be invoked
    after a test method completes.

    :   **Returns:**: All the methods that should be invoked after a test method completes.

---


### getBeforeClassMethods

```
ITestNGMethod[] getBeforeClassMethods()
```

:   Return all the methods that should be invoked
    after the test class has been created and before
    any of its test methods is invoked.

    :   **Returns:**: All the methods that should be invoked after the test class has been created and before any of its test methods is invoked.

---


### getAfterClassMethods

```
ITestNGMethod[] getAfterClassMethods()
```

:   Returns all the methods that should be invoked
    after all the tests have been run on this class.

    :   **Returns:**: All the methods that should be invoked after all the tests have been run on this class.

---


### getBeforeSuiteMethods

```
ITestNGMethod[] getBeforeSuiteMethods()
```

:   Returns All the methods that should be invoked
    before the suite is run.

    :   **Returns:**: All the methods that should be invoked before the suite is run.

---


### getAfterSuiteMethods

```
ITestNGMethod[] getAfterSuiteMethods()
```

:   Returns all the methods that should be invoked
    after the suite has run.

    :   **Returns:**: All the methods that should be invoked after the suite has run.

---


### getBeforeTestConfigurationMethods

```
ITestNGMethod[] getBeforeTestConfigurationMethods()
```

:   Returns all @Configuration methods that should be invoked before any others in the
    current test.

    :   **Returns:**: all @Configuration methods that should be invoked before any others in the current test.

---


### getAfterTestConfigurationMethods

```
ITestNGMethod[] getAfterTestConfigurationMethods()
```

:   Returns all @Configuration methods that should be invoked last before any others
    in the current test.

    :   **Returns:**: all @Configuration methods that should be invoked last before any others in the current test.

---


### getBeforeGroupsMethods

```
ITestNGMethod[] getBeforeGroupsMethods()
```

:   Returns all @Configuration methods that should be invoked before certain groups.

    :   **Returns:**: all @Configuration methods that should be invoked before certain groups.

---


### getAfterGroupsMethods

```
ITestNGMethod[] getAfterGroupsMethods()
```

:   Returns all @Configuration methods that should be invoked after certain groups.

    :   **Returns:**: all @Configuration methods that should be invoked after certain groups.


---


|  |  |  |
| --- | --- | --- |
| **Overview** \| **Package** \| **Class** \| **Tree** \| **Deprecated** \| **Index** \| **Help** | |  |
| **PREV CLASS**   **NEXT CLASS** | **FRAMES**    **NO FRAMES**     **All Classes** |  |
| SUMMARY: NESTED \| FIELD \| CONSTR \| METHOD | DETAIL: FIELD \| CONSTR \| METHOD |  |


---
